# Is it possible to store spotted wolffish (*Anarhichas minor*) sperm by refrigeration?

**DOI:** 10.1007/s10695-020-00820-w

**Published:** 2020-06-02

**Authors:** W.A. González-López, D.M. Patel, N. Duncan, J. Beirão

**Affiliations:** 1 Faculty of Biosciences and Aquaculture, Nord University, NO-8049 Bodø, Norway; 2 IRTA Sant Carles de la Rápita, 43540 Sant Carles de la Rápita, Tarragona, Spain

**Keywords:** Spotted wolffish, Sperm short-term storage, Sperm quality, Urine contamination, Energy stores

## Abstract

Spotted wolffish *Anarhichas minor* reproduction in captivity is dependent on in vitro fertilization. However, it is often challenging to acquire sufficient fresh sperm to fertilize the eggs that are obtained. In this study, we evaluate the possibility to store spotted wolffish sperm by refrigeration. Spotted wolffish sperm has the particularity that is already motile on stripping, and currently it is not possible to immobilize and reactivate. Thus, sperm refrigeration protocols should focus in extending this motility period that usually lasts up to 2 days. In a first experiment, we evaluated the possibility that the motility period of the sperm was limited by contamination with urine. The urea concentration in the sperm obtained both by stripping (17.10 ± 1.98 mg/dL) and directly from the testis (12.59 ± 2.37 mg/dL) was similar (*p* > 0.05), which indicate that the sperm collection method used avoid contamination with urine. Afterwards, we tested the possibility that the sperm motility period was limited by energy stores. The ATP concentration (initial value 5.65 ± 0.86 nmol/10^9^ cells) remained stable (*p* = 0.099) during 30 h after sperm collection, and similar values (*p* = 0.329) were recorded at end of sperm storage in both diluted (3.88 ± 1.35 nmol/10^9^ cells) and undiluted samples (4.76 ± 1.08 nmol/10^9^). This indicates that the low intracellular ATP consumption, derived from the slow sperm motility, can probably be compensated rapidly enough by mitochondrial synthesis of ATP in the spotted wolffish sperm. In both experiments, diluted sperm kept higher percentage of motile cells during the storage time.

## Introduction

The spotted wolfish *Anarhichas minor* has emerged as a potential species for marine cold-water aquaculture in Canada, Iceland, Sweden and Norway. This aquaculture potential is based on good growth rates, high fillet yield, few disease problems, relatively high market price and consumer acceptance (Falk-Petersen et al. 1999; Le François et al. 2002; Foss et al. 2004). In Norway, the species is already reared on a commercial scale, but production volumes are low and quite unstable. The main reason for this instability has been attributed to problems to provide sufficient good quality eggs. In captivity, the spotted wolffish reproductive behaviour is disrupted, and fertilization must be conducted in vitro (Falk-Petersen et al. 1999; Beirão and Ottesen 2018). However, males produced relatively low sperm volume with a low concentration and motility compared with other marine fish (Le François and Archer 2007; Beirão and Ottesen 2018). In addition, the sperm that is already motile at stripping remains motile from several hours up to 2 days, but is inactivated by contact with seawater (Kime and Tveiten 2002). Thus, sperm storage procedures, through cryopreservation, have been suggested as methods to ensure the availability of good quality sperm when required (Le François et al. 2008; Gunnarsson et al. 2009; Santana et al. 2020).

Cryopreservation protocols require access to liquid nitrogen, specific material and chemicals, trained personnel and in small hatcheries, as is the case for spotted wolffish, are logistically difficult to implement. In addition, cryopreservation procedures are often used for long-term sperm storage (Cabrita et al. 2010). Short-term storage of sperm by refrigeration, on the other hand, is a simple and inexpensive procedure relatively easy to implement in the hatchery environment (Bobe and Labbé 2008; Contreras et al. 2019). Sperm refrigeration protocols have been tested in several species and can be a useful tool to assist in artificial fertilization programmes, synchronization in availability of gametes and transport of gametes between different facilities (Contreras et al. 2019; Beirão et al. 2019). Short-term storage is especially relevant when the sperm volume obtained is limited (Bobe and Labbé 2008). Thus, in this study, we test the possibility of sperm refrigeration as an option to secure spotted wolffish sperm availability for a few days.

Whilst sperm refrigeration is an attractive option for spotted wolffish, it presents challenges as the sperm is already motile at stripping, and it is currently not possible to immobilize and reactivate wolffish sperm. Therefore, sperm refrigeration protocols must provide the conditions to ensure the sperm motility period extends over the duration of the required storage period. Furthermore, spotted wolffish sperm is collected by stripping (Le François and Archer 2007), which increases the risk of contamination of semen with urine, as observed in other fish species (Sarosiek et al. 2016; Król et al. 2018). As an example, in both pikeperch *Sander lucioperca* and turbot *Psetta maxima*, a higher urine contamination was exhibited in the sperm collected by stripping compared with sperm collected with a catheter (Dreanno et al. 1998; Sarosiek et al. 2016). This contamination with urine is usually linked to a reduction in sperm quality and storability (Król et al. 2018; González-López et al. 2019). A decrease in the percentage of sperm motility and sperm velocity parameters (VCL and VSL) in pikeperch was observed due to urine contamination (Sarosiek et al. 2016). In turbot, higher urine concentration caused a delay in the motility initiation (Dreanno et al. 1998). Damage to sperm motility rate in sperm samples from Senegalese sole *Solea senegalensis* and Eurasian perch *Perch fluviatilis* stored for 24 h was related to the urine concentration in sperm (Król et al. 2018; González-López et al. 2019).

In the present study, two experiments were conducted in order to test methods for the short-term refrigerated storage of spotted wolffish sperm. In the first experiment, the degree of urine contamination in relation to a stripping method to avoid urine and short-term refrigerated storage of the sperm was evaluated. In the second experiment, the importance of the energy reserves on sperm quality and short-term refrigerated storage was assessed.

## Material and methods

### Samples collection

This study was carried on with the licence (No A08, 017) from the Norwegian Food Safety Authority (Mattilsynet) attributed to the Faculty of Bioscience and Aquaculture, Nord University, to perform experiments on animals. Sperm samples were obtained from the spotted wolffish producer AMINOR AS (Halsa, Nordland, Norway) during the reproductive season (January–March 2019). Ten-year-old adult breeders were kept all year round in 1600 L rectangular tanks, water depth 0.4 m, with an open flow through system under natural temperature and photoperiod conditions (66° 74′ N, 13° 51′ E). Oxygen measurements of the outgoing water were kept over 80%. The fish were fed with pellets (Vitalis CAL and Vitalis REPRO, Skretting).

Individuals were anaesthetised with 500 ppm tricaine methanesulfonate (MS-222, Sigma-Aldrich) for 5 min. The sperm collection procedure follows Beirão and Ottesen (2018). After the release of most urine by pressing the abdominal area, a gentle massage was applied from the lateral region of the abdomen where the testes and the sperm ducts are located towards the urogenital pore to obtain sperm samples. The sperm samples (1.0 mL for experiment 1 and 1.5 mL for experiment 2, see below) were collected in a plastic Pasteur pipette attached to the urogenital pore. The samples were kept in a refrigerator (2 °C) until analysis. Urine samples (2 mL) were obtained by applying pressure to the urinary bladder. The urine samples were collected into a 15-mL falcon tube and stored at − 20 °C until analysis of the urea concentration.

### Sperm quality assessment

#### Motility parameters

Percentage of motile cells and curvilinear velocity (VCL) (μm/s) were evaluated. Initially, 1 μL of the sperm samples was diluted 1:10 in solution previously developed by Smith and Ryan (2010) for internal fertilizing fish (207 mM NaCl, 1.3 mM CaCl_2_, 0.41 mM MgSO_4_, 5.4 mM KCl, 0.49 mM MgCl_2_ and 10 mM Trizma) with the addition of 1% BSA, and the pH was adjusted to 7.5. A preliminary trial demonstrated that the solution developed by Smith and Ryan (2010) was an adequate extender for refrigerated wolffish sperm storage compared with the solution previously developed by Kime and Tveiten (2002). A 2 μL drop of the diluted sample was placed between a coverslip and a slide, and videos were recorded with the CASA system SCA 6.2 (Microptic, Barcelona, Spain). Images were recorded using a digital camera (Basler acA1300-200uc, Ahrensburg, Germany) attached to an optical phase-contrast microscope (Nikon Eclipse Ci, Tokyo, Japan) with × 10 negative phase contrast objective, with a stage temperature controller set to 6 °C (Linkam T95-PE, Tadworth, UK). Samples were analysed in triplicate.

#### Cell concentration

The cell concentration in each sperm sample was calculated from counts of diluted sperm sample. A sample of 10 μL of fresh sperm was diluted 1:500 with Smith and Ryan (2010) extender, and 10 μL of this dilution was placed into a Neubauer counting chamber. The sample was observed under the phase-contrast microscope with a × 10 objective. For each sample, the count was made in triplicate.

#### pH and osmolality

The pH was determined in the seminal plasma by the colorimetric method using pH indicator strips (Hydrion, Sigma-Aldrich), with a detection range between 5 and 9 pH. The osmolality (mOsm/kg) was determined from 10 μL seminal plasma using the freezing point depression osmometer (Fiske One-Ten, Fiske® Associates), and each sample was measured in duplicate.

#### ATP and glucose concentration

A 100 μL sample of semen was used to determine the ATP concentration. Initially, 100 μL of EDTA solution with TCA (4%) was added to 100 μL semen and mixed with a vortex mixer before being centrifuged (10,000 × g for 10 min at 4 °C). From this centrifuged suspension, 100 μL of supernatant was collected and added to 500 μL Sorensen buffer (adjusted pH 7.8) before being stored in a 1.5-mL centrifuge tube at − 20 °C for further analysis. The ATP concentration was determined by using the Adenosine 5′-triphosphate (ATP) Bioluminescent Assay kit (FLAA, Sigma-Aldrich) in accordance with the manufacturer’s instructions. The samples luminescence was read in a FLUOstart Optima plate reader (BMG LABTECH, Ortenberg, Germany) in a 96-well white plate. The ATP concentration was calculated and expressed in nanomoles per 10^9^ cell.

The glucose concentration (μg/mL) was assessed with the Glucose (GO) Assay kit (GAGO-20, Sigma-Aldrich) following the manufacturer’s instructions. Seminal plasma samples diluted 5× were processed with the kit, and the absorbance was read in a 96-well transparent plate at 540 nm in the FLUOstar Optima plate reader (BMG LABTECH).

#### Protein analysis

The total protein concentration (mg/mL) was evaluated using the Invitrogen Qubit® Protein Assay Kit and Qubit™ Fluorometer (Thermo Fisher Scientific). One dimensional gel electrophoresis was used to separate the proteins in the seminal plasma according to molecular weight. The sample for the gel electrophoresis was prepared by diluting (1:1) the seminal plasma in 0.7% NaCl solution. The diluted sample (20 μl) was mixed with equal amount of 1× Laemmli buffer (Bio-Rad, USA). The mixture was vortexed for 30 s and incubated for 5 min at 95 °C; after incubation, 30 μl of each sample was loaded in the gel (12.5% polyacrylamide gel) together with the protein ladder (Precision Plus Protein™ Kaleidoscope prestained protein standards, Bio-Rad, USA). The gel was ran at 200 V until the dye front reached the bottom of the gel (~ 30 min). After the electrophoresis, the bands were stained using Coomassie Brilliant Blue R-250 (Bio-Rad, USA) for 2 h. After 2 h, the destaining solution (40% methanol and 10% glacial acetic acid) was used on the gel until the background was clear (~ 2 h). The gel was then rinsed in miliQ water, and the image was documented using ChemiDoc™ MP imaging system (Bio-Rad, USA). The analysis of the bands was performed using the Image Lab Software (Bio-Rad).

#### Urea concentration

The urea concentration was measured in both seminal plasma and urine samples with the Urea assay kit (KA 1652, Abnova, Denmark) following the manufacturer’s instructions. The samples, processed with the kits reagents, were placed into a 96-well plate and incubated for 20 min at room temperature. The plate was read in the FLUOstar Optima plate reader (BMG LABTECH) at 520 nm. The readings were transformed and expressed in units of milligrams per deciliter.

### Experiment 1: stripping method, urine contamination and short-term refrigerated storage of sperm

To evaluate the degree of urine contamination on sperm quality and the effect on storability, sperm and urine samples (*n* = 9) were collected from different males. The sperm samples were divided in four aliquots. The first aliquot, with 15 μL, was used to assess cell concentration and percentage of motility and VCL within 10 min of sperm collection. The second aliquot, with 100 μL, was diluted (D) 1:2 (final volume 300 μL) in the solution developed by Smith and Ryan (2010). The third aliquot, with 300 μL, was kept undiluted (UD). Both the second (D) and third aliquot (UD) were stored in an incubator at 2 °C to assess the percentage of motile cells and VCL at different times after collection (5, 10, 20, 30, 40 and 50 h). The fourth aliquot, with 400 μL, was centrifuged for 10 min at 300×*g* and room temperature to collect the seminal plasma. In addition, seminal plasma was obtained from sperm obtained directly from the testes of two sacrificed males. The seminal plasma was stored at − 20 °C until its analysis in the laboratory for pH, osmolality, urea and total protein.

### Experiment 2: effect of energy reserves on short-term refrigerated storage of sperm

To assess the importance of ATP and glucose reserves on sperm storability, sperm samples (*n* = 8) were separated in five aliquots. The first aliquot of 15 μL was used to assess cell concentration and percentage of motility and VCL within 10 min of sperm collection. The second aliquot of 300 μL was diluted (D) 1:1 (final volume 600 μL) in the Smith and Ryan (2010) solution. The third aliquot of 600 μL was undiluted (UD). Both the second and the third aliquots were stored in an incubator at 2 °C and assess at different times (5, 10, 20 and 30 h) for percentage of motility, VCL, ATP and glucose. Glucose was only measured in UD samples. The fourth aliquot of 100 μL of fresh sperm was processed to analyse the ATP content. The fifth aliquot of 450 μL was centrifuged for 10 min at 500×*g* and 4 °C, and the seminal plasma was collected and placed in 1.5-mL centrifuge tubes and frozen at − 20 °C until analysis of pH, osmolality, glucose, total protein and proteins based on their molecular weight.

### Statistical analysis

The data were analysed with IBM SPSS Statistics 20 and expressed as mean ± one standard error of the mean (SEM). The data were analysed for normality using the Shapiro-Wilks test. In both trials, correlations between percentage of motility, VCL and the different parameters measured were analysed with a Pearson’s correlation. For each experiment (1 and 2), two General Linear Models (repeated measures) were performed to detect possible differences between D and UD treatments, one for the percentage of motility and the other for VCL, during the storage time. An additional General Linear Model (repeated measures) was performed to evaluate the ATP parameter between D and UD treatments and over time. Urea concentration measured in urine and seminal plasma collected from the testes and by stripping was compared through a one-way ANOVA and a Tukey post-hoc. Differences were considered significant for *p* < 0.05.

## Results

### Experiment 1: stripping method, urine contamination and short-term refrigerated storage of sperm

**Fig. 1:**
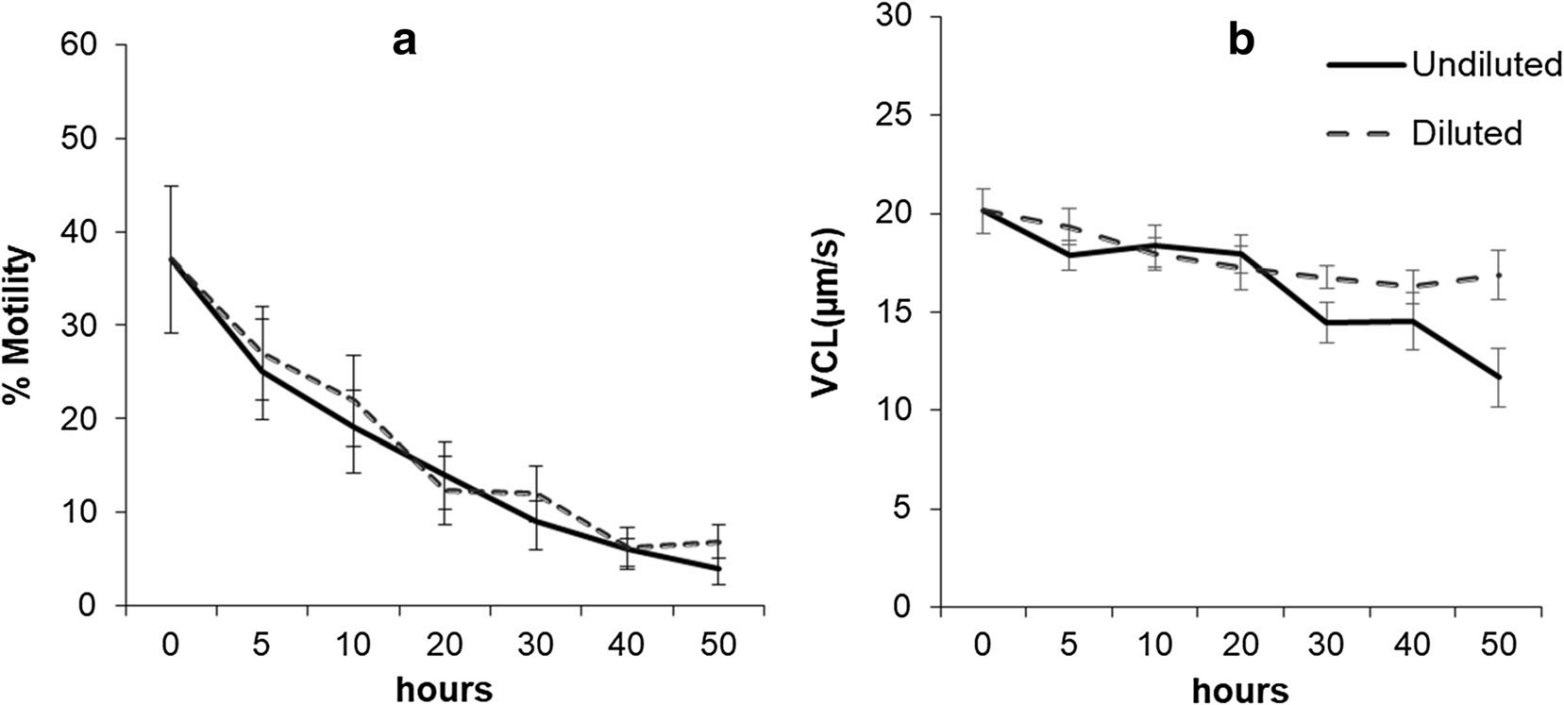
**a** Percentage of sperm motility and **b** curvilinear velocity (VCL) (μm/s) between diluted (D) and undiluted (UD) spotted wolffish sperm samples during refrigerated storage in the experiment 1. Values represent the mean ± SEM (*n* = 9)

In the first experiment, the initial sperm motility was 36.4 ± 7.89%, and VCL was 20.11 ± 1.14 μm/s, with a range between 7.35 and 70.81% and from 12.63 to 25.22 μm/s, respectively. For the UD samples, there was a significant decrease (*p* = 0.042) during the storage time, from 0 to 50 h, in the percentage of motile cells, whilst D samples did not show any difference (*p* = 0.065) between 0 and 50 h (Fig. 1a). However, at 50 h after storage, there was no significant difference (*p* = 0.434) between the percentage of motility in D (6.88 ± 1.76%) and in UD (4.39 ± 1.08%). For the VCL, there were no significant differences for D and UD over time or between the two treatments at 50 h (*p* ≥ 0.063) (Fig. 1b).

**Fig. 2:**
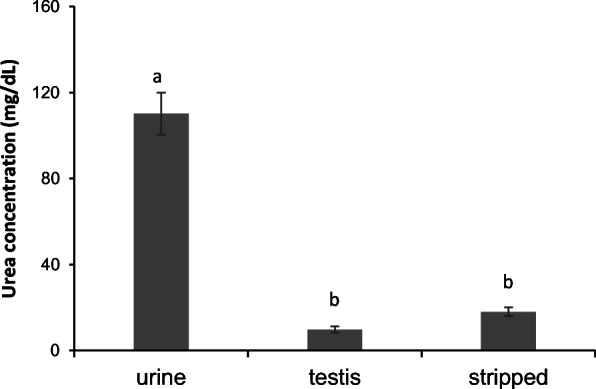
Differences in urea concentrations (mg/dL) in spotted wolffish pure urine samples and seminal plasma obtained from testes sperm and by stripping. Different letters stand for significant differences between group of samples as detected with a one-way ANOVA (*p* < 0.05). Values represent the mean ± SEM

The urea concentration was significantly higher in the urine (110.18 ± 9.83 mg/dL) compared with the stripped sperm seminal plasma (17.10 ± 1.98 mg/dL). No differences in urea concentration were observed between the seminal plasma of stripped sperm and the seminal plasma from testis sperm (Fig. 2). The urea concentration in the stripped samples was correlated with percentage of motile cells at 50 h after storage (*R* = 0.752; *p* = 0.031). The urine samples pH was 5.61 ± 0.13 and the osmolality 299.89 ± 4.94 mOsm/kg. Neither the seminal plasma pH 6.37 ± 0.06 nor osmolality 319.56 ± 9.94 mOsm/kg was correlated with the sperm motility parameters, whilst the total protein concentration (0.76 ± 0.16 mg/ml) was negatively correlated with VCL at 5, 20, 30 and 40 h of storage in the diluted samples (*R* = − 0.690, *p* = 0.040; *R* = − 0.774, *p* = 0.024; *R* = − 0.802, *p* = 0.009; *R* = − 0.755, *p* = 0.030, respectively).

### Experiment 2: effect of energy reserves on short-term refrigerated storage of sperm

**Fig. 3:**
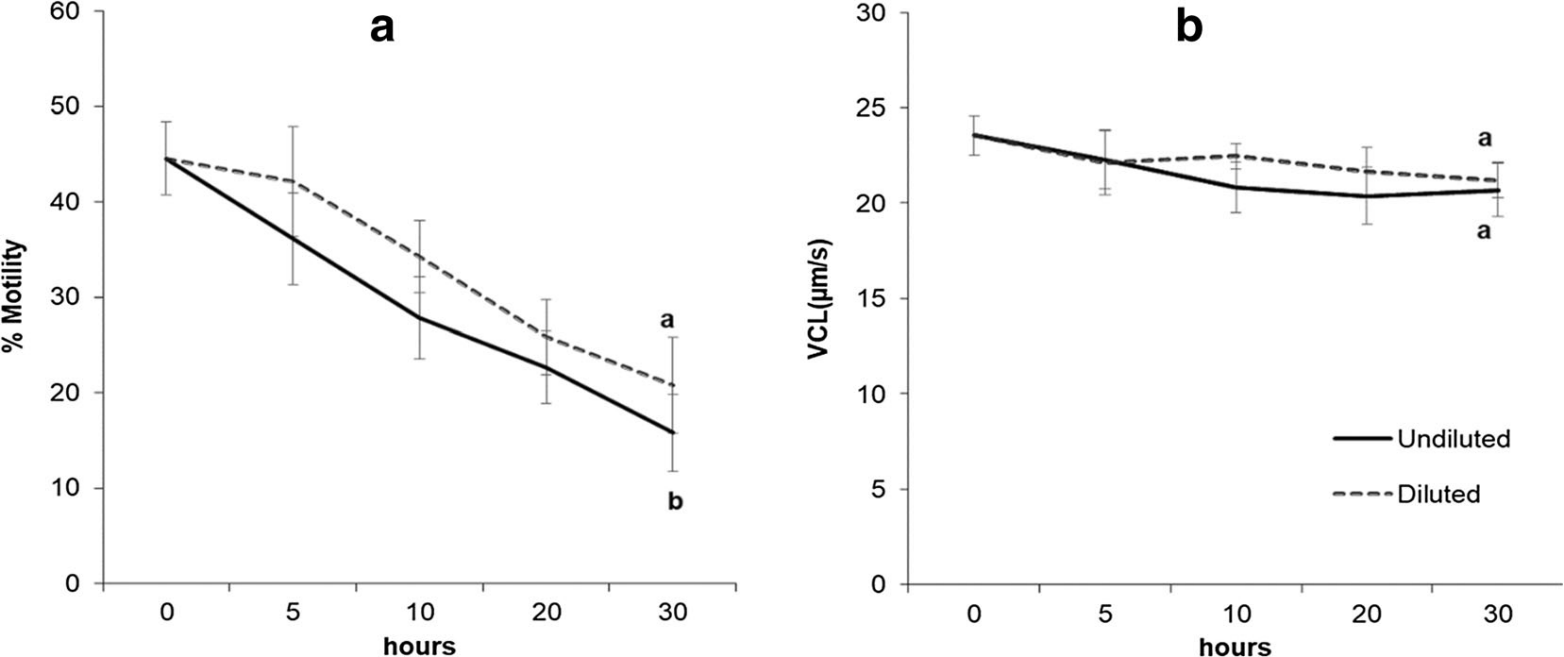
**a** Percentage of sperm motility and **b** curvilinear velocity (VCL) (μm/s) between diluted (D) and undiluted (UD) spotted wolffish sperm samples during the refrigerated storage in the experiment 2. Values represent the mean ± SEM (*n* = 8). Different lowercase letters indicate significant differences between samples (D) and (UD) at the end of short-term sperm storage

The initial sperm quality values (44.56 ± 3.85% motile cells, VCL 23.55 ± 1.04 μm/s, seminal plasma pH 6.11 ± 0.12 and osmolality 314.25 ± 4.37 mOsm/kg) observed in the experiment 2 were similar to experiment 1. However, significant differences were found for the percentage of motile cells between the D (20.83 ± 5.02%) and UD (15.79 ± 4.05%) treatments (*p* < 0.05) at 30 h of storage (Fig. 3a). On the other hand, as in experiment 1, the sperm velocity (VCL) was not significantly different between the diluted sperm samples (21.21 ± 0.95 μm/s) and the undiluted samples (20.68 ± 1.40 μm/s) at 30 h (Fig. 3b).

**Fig. 4:**
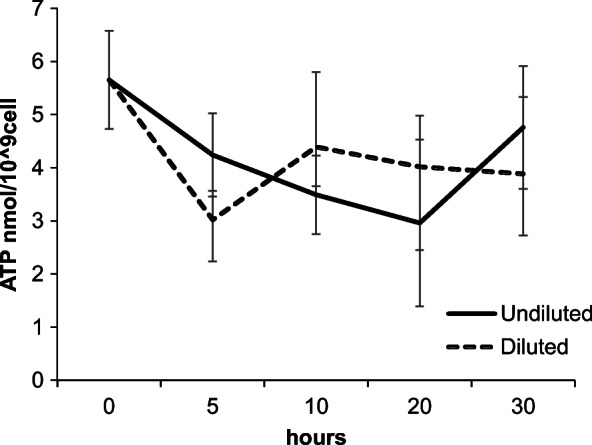
Mean ATP values observed (nmol/10^9^ cells) in diluted (D) and undiluted (UD) spotted wolffish sperm samples during the refrigerated storage in experiment 2. Values represent the mean ± SEM (*n* = 8)

**Fig. 5:**
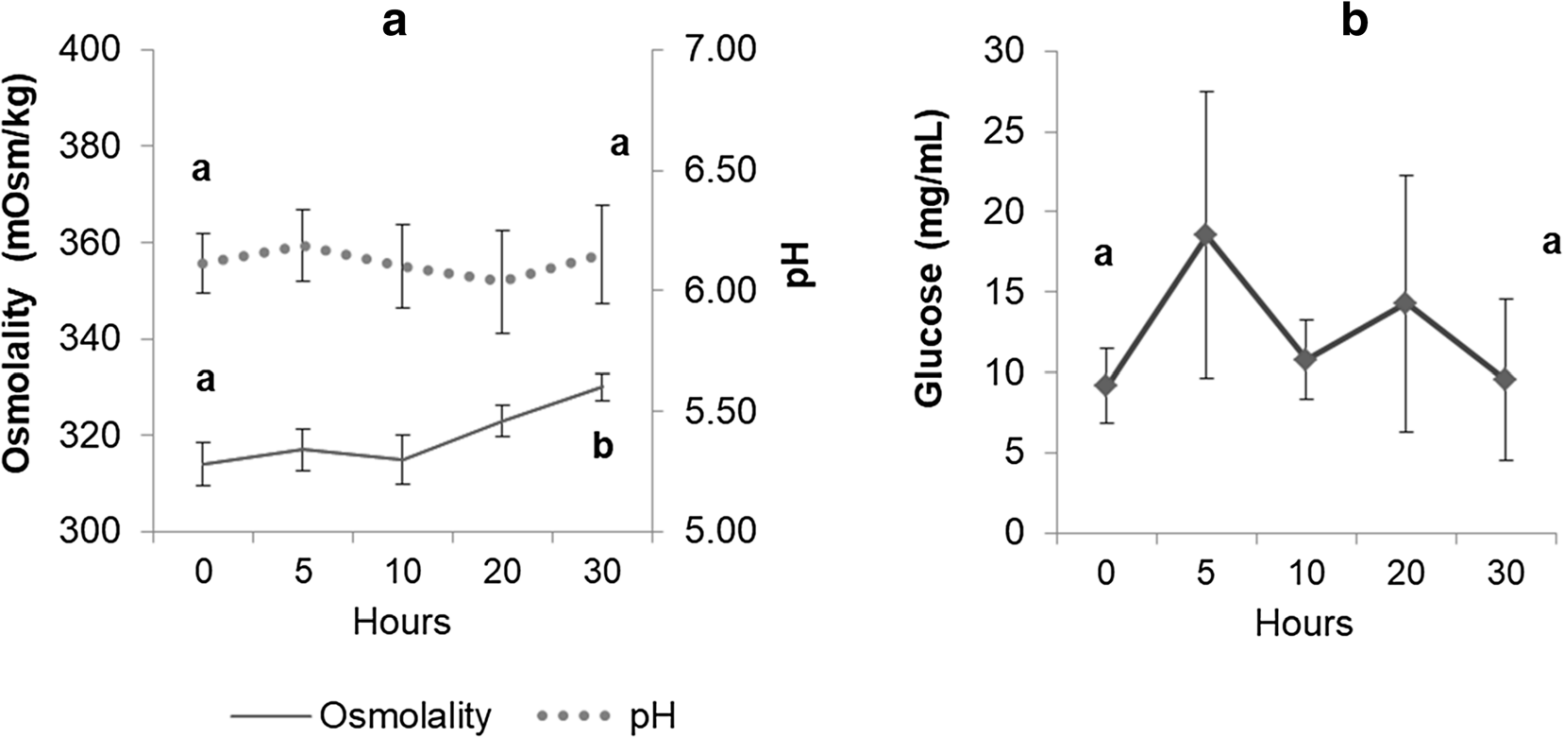
Osmolality (mOsm/kg), pH (**a**) and glucose (mg/mL) values (**b**) in seminal plasma of undiluted (UD) spotted wolffish sperm during the refrigerated storage time in experiment 2. Values represent the mean ± SEM (*n* = 8). Different lowercase letters indicate significant differences between the initial value and the final value at the end of short-term sperm storage

The initial ATP values (5.65 ± 0.86 nmol/10^9^ cells) were not significantly affect (*p* = 0.099) by the storage period, and at 30 h the values were similar (*p* = 0.329) in both D (3.88 ± 1.35 nmol/10^9^ cells) and UD (4.76 ± 1.08 nmol/10^9^ cells) samples (Fig. 4). Likewise, the pH (initial value 6.11 ± 0.12; final value 6.15 ± 0.20) and glucose (initial value 9.13 ± 2.32 μg/mL; final value 9.51 ± 4.99 μg/mL) values also remained stable (*p* = 0.981 and *p* = 0.732, respectively) during the storage time (Fig. 5a, b), whilst osmolality had a significant increase (*p* = 0.039) through the storage period (initial value 314.52 ± 4.37 mOsm/kg; final value 330.62 ± 2.82 mOsm/kg). In addition, no correlation was observed between ATP or the glucose and the sperm motility parameters (percentage of motile cells and VCL).

The protein concentration was similar to the first experiment (0.76 ± 0.23 mg/mL). Fourteen protein bands were detected. The bands with molecular weights of 80, 70, 60, 20 and 15 kDa each represented more than 10% of the protein amount detected in the seminal plasma (Table 1). The 150 kDa protein band, although absent in three of the seminal plasma samples, had negative correlation with both percentage of motility (*R* = − 0.966; *p* = 0.007) and VCL (*R* = − 0.964; *p* = 0.008) at the time 0 h. The 80 kDa protein band was also negatively correlated with VCL at 30 h (*R* = − 0.777; *p* = 0.023). The remaining protein bands with higher percentage of protein were not correlated with either the percentage of motile sperm or VCL.

**Table 1:** Protein bands according to the molecular weight found in seminal plasma of spotted wolffish. Values in the right column represent the mean percentage (*n* = 8)

Proteins (kDa)	Mean (%)
80	14.86
70	20.80
60	26.56
20	11.73
15	18.08

## Discussion

Refrigeration of sperm could be a more practical approach for storage of spotted wolffish sperm for short periods (days) compared with the logistically complex and expensive sperm cryopreservation protocols currently used. In both short-term storage experiments, after 30 h of refrigerated storage, the samples maintained percentage of sperm motilities between 10 and 20% and sperm velocities close to 20 μm/s. These sperm quality parameters exhibited similar values to the cryopreservation method used in spotted wolffish (Santana et al. 2020). After cryopreservation, Santana et al. (2020), depending on the spermatozoa: egg ratio could obtain values above 80% fertilization. This indicates that the refrigeration method described in the present study can be used to store sperm for 1–2 days, and diluted sperm samples retained higher percentage of motile sperm throughout the storage period.

After the storage time, the diluted sperm percentage of motility was only significantly higher in the second experiment. Nonetheless, in the first experiment, diluted samples retain similar percentage of motile cells through the storage, whilst undiluted had a significant decrease. The large range of initial samples quality in the first experiment could partly explain the difficulty to detect significant differences in storability in terms of percentage of motile sperm. Most authors report an improvement in samples storability by the use of an extender solution (Gallego et al. 2013; Santos et al. 2018; González-López et al. 2019). The use of these extender solutions is beneficial for several reasons such as reduces the sperm density and thus improves the oxygen supply, controls pH through the use of buffers such as Tris in our case, prevents the dehydration of the cells and decreases the harmful effect of urine contamination (reviewed by Contreras et al. 2019; Beirão et al. 2019). However, in most cases reported the extender resembles the seminal plasma and is designed to keep the sperm in quiescent immotile state until activation by contact with fresh (freshwater species) or seawater (marine species) (Contreras et al. 2019), which is not the case of spotted wolffish. Few exceptions exist for internal fertilizing species, such as guppy *Poecilia reticulata* (Sun et al. 2010), where the sperm was continuously motile in the extender solution. However, in these livebearers aquarium fish, such as the guppy (Sun et al. 2010) or the green swordtail *Xiphophorus helleri* (Yang et al. 2006), it was possible to recover sperm motility after inhibition with a low or high osmolality. In spotted wolffish, preliminary trials indicate that it is not possible to recover motility after exposing to higher or low osmolality inhibiting solution (data not shown).

Several authors have correlated the urea levels with contamination by urine and decrease sperm quality (Dreanno et al. 1998; Fauvel et al. 2012; Król et al. 2018; González-López et al. 2019). Nonetheless, in our study, the level of urea concentration in seminal plasma was similar both in the samples obtained from testis or by stripping and significantly lower than in urine. In addition, at the time of collection (0 h), there was no correlation between urea concentration and sperm quality parameters indicating that the low concentrations of urea in the present study did not affect sperm quality. Other studies have found that relatively low urine contamination does not necessarily affect the sperm quality, as was observed in Atlantic halibut *Hippoglossus hippoglossus* (Babiak et al. 2006) and European seabass *Dicentrarchus labrax* (Fauvel et al. 2012). In addition, seminal plasma pH and osmolality values are usually affected by contamination with urea. Which does not seem to be the case of our study, the urine pH and osmolalilty values were lower than the seminal plasma values (5.61 ± 0.13 vs 6.37 ± 0.06 for pH and 299.89 ± 4.94 mOsm/kg vs 319.56 ± 9.94 mOsm/kg for urine and seminal plasma, respectively), and neither of them was correlated with the urea concentration in the seminal plasma. Whereas the osmolality values were similar to the ones previously reported by Kime and Tveiten (2002) of 310–330 mOsm/kg, the seminal plasma pH obtained in our study was higher (pH of 4.8–7.7 in Kime and Tveiten 2002). This could indicate that part of the samples collected by Kime and Tveiten (2002) had some urine contamination. According to the methods described, these authors collected the sperm by direct pressure in the belly whereas we applied the sperm collection method recommended by Beirão and Ottesen (2018). In this case, the urine is cleared before, and only after the sperm is collected. Thus, the procedures of sperm collection used in this work should be enough to avoid semen pollution with urine in spotted wolffish. However, the level of urea was positively correlated with percentage of motility assessed at 50 h after storage in undiluted samples, which indicates that the urea measured in the seminal plasma is more related with the normal sperm protein metabolism. It would appear that higher numbers of motile sperm for a long time increased the urea concentration compared with samples with lower numbers of motile sperm.

Unexpectedly, the ATP concentration remained stable along the storage time even though the percentage of motile sperm dropped from 44.56 ± 3.85% to 20.83 ± 5.02% in diluted sperm and 15.79 ± 4.05% in undiluted sperm. To our knowledge, all studies so far that have looked at ATP during sperm storage by refrigeration have observed a decrease in its concentration along the time (e.g. meagre *Argyrosomus regius* (Santos et al. 2018), rainbow trout *Oncorhynchus mykiss* (Bencic et al. 1999)). However, in most studies sperm is kept in a quiescent immotile state, whereas the sperm of spotted wolffish is motile on stripping. Thus, different conditions for refrigerated sperm storage need to be considered. Moreover, the initial ATP concentration of 5.65 ± 0.86 nmol/10^9^ cells is relatively low compared with other fish species, usually in the range of 4–24 nmol/10^8^ cells (revised by Dzyuba et al. 2017). In most species with external fertilization, the sperm activation occurs by an osmotic or ionic change; the motility period is very short (1–2 min) and at high velocity (> 100 μm/s), which leads to rapid decrease in ATP concentration (Dzyuba et al. 2017; Kowalski and Cejko 2019). Indeed, the intracellular ATP content is usually related with the duration of the sperm motility period and sperm velocity, and the completion of the motility period is partly caused by low intracellular ATP (Dreanno et al. 1999; Dzyuba et al. 2017). In contrast, spotted wolffish is characterized by low velocity and long period of sperm motility as observed in the present study and reported by Kime and Tveiten (2002). In addition, the spermatozoa are characterized by a large midpiece with high number of mitochondria, as observed in the closely related common wolffish *Anarhichas lupus* (Pavlov et al. 1997). Thus, the low velocity, and thus low intracellular ATP consumption, can probably be compensated rapidly enough by mitochondrial synthesis of ATP via respiration for the 1 to 2 days period of motility usually observed.

Similar to the ATP, also the seminal plasma glucose values remained constant during the storage time (30 h). The initial glucose concentration obtained in this work (9.13 ± 2.32 μg/mL) was higher than the values observed in other marine species, such as gilthead seabream *Sparus aurata* 6.13 ± 4.68 μg/mL, total value for semen with cells, (Lahnsteiner et al. 2010) or turbot 0.92 ± 0.20 μg/mL (Dreanno et al. 1998). As discussed by Yang and Tiersch (2009), different species use different sources of energy for sperm motility. Whereas in some species sperm cells are able to use exogenous sources as carbohydrates, in other species the sperm cells apparently use exclusively endogenous sources of energy. As an example, both in African catfish *Clarias gariepinus* (Zietara et al. 2004) and in abant trout *Salmo trutta abanticus* (Hatipoglu and Akcay 2010), the addition of energetic substrates, such as glucose, helped maintain the ATP levels and improved motility during the storage time. On the other hand, in medaka *Oryzias latipes* the sperm motility is not affected by the presence or absence of glucose in the medium (Yang and Tiersch 2009). The fact that spotted wolffish seminal plasma glucose levels remain constant during the 30 h storage period does not mean it cannot use exogenous sources for the maintenance of the ATP levels, since it could be using other sources. Thus, this question should be further explored to aid in the development of refrigerated storage protocols for the sperm of this species.

The protein concentration in both experiments, compared with other marine species, was lower than in Atlantic halibut (6.4 to 19.4 mg/mL) (Mommens et al. 2008), but in similar range to Atlantic cod *Gadus morhua* (0.78 to 1.05 mg/mL) (Butts et al. 2011). The proteins contained in the seminal plasma are mostly involved in sperm protection, but each of them has different roles such as sperm maturation, cell death, respiration, lipid metabolism, energy production and motility (reviewed by Ciereszko et al. 2016). Indeed, most authors link the increase in specific proteins with improved sperm quality. For example, in European eel *Anguilla anguilla* samples with proteins with molecular weight less than 50 kDa were linked to increase in the sperm motility (Peñaranda et al. 2010). However, in our work most of the correlations we observed between the proteins (total and protein bands) and sperm quality parameters were negative correlations. Spotted wolffish do not release sperm naturally in captivity, and thus the sperm obtained by stripping could be overripe and these proteins be related with cell death. However, in order to make such conclusion, a more detailed proteomic study will be needed using for example markers for proteolytic activity (e.g. Mommens et al. 2008) or a 2-D gel analysis (e.g. Zilli et al. 2014) for protein identification. Nonetheless, the analysis of the proteins in the present experiment opens the possibility to use proteomic indicators for sperm selection.

In this work, we present important data that will help in the future development of a refrigerated storage protocol for spotted wolffish sperm. First, wolffish sperm can be stored for 1–2 days, the dilution of the sperm in a medium improves its storability, and thus the composition of this medium should be further explored. Secondly, using the reported sperm collection method (Beirão and Ottesen 2018) urine contamination was avoided and hence did not affect the sperm storability. Finally, the sperm motility period is not limited by the ATP levels that remain stable at least for the first 30 h; however, the energy source used to maintain these ATP levels is not clear.
